# Case report: Dupilumab: a promising treatment option for adult linear IgA bullous dermatosis with severe pruritus

**DOI:** 10.3389/fimmu.2024.1409556

**Published:** 2024-08-05

**Authors:** Li Wang, Jing Peng, Jinbo Chen

**Affiliations:** Department of Dermatology, Wuhan No. 1 Hospital, Wuhan, China

**Keywords:** linear IgA bullous dermatosis (LABD), dupilumab, pruritus, dapsone, HLA-B*1301

## Abstract

Linear IgA bullous dermatosis (LABD) is an acquired autoimmune subepidermal blistering disorder. Diagnosis always relies on skin pathology and direct immunofluorescence (DIF), with typical linear deposits of IgA along the basement membrane zone (BMZ). The typical clinical manifestation is tense bullae arranged like the “string of pearls” companied with severe pruritus. Dapsone is often considered first-line therapy for LABD, and it is necessary to test the HLA-B*1301 gene to prevent the occurrence of dapsone-induced hypersensitivity syndrome (DHS). Here we report a case of LABD resistant to corticosteroid and sulfasalazine, while waiting for HLA-B*1301 gene test results, dupilumab was used to control severe pruritus.

Linear IgA bullous dermatosis (LABD) is a rare autoimmune vesicobullous disease and its clinical appearance may be polymorphic forms characterized by erythematous papules, bullae, vesicles, and erosion. The most characteristic feature are bullae arranged in a circular pattern, called ‘cluster of jewels’ or ‘string of pearls’. The gold standard for diagnosis is direct immunofluorescence (DIF), which shows linear deposition of IgA along the basement membrane zone (BMZ) (1). The first-line therapy should be dapsone and steroids (2, 3), Sulfasalazine is also effective in LABD (2). Dapsone-induced hypersensitivity syndrome (DHS) is a life-threatening adverse drug reaction, which was found to be related to HLA-B*1301. For patient safety, genetic screening for HLA-B*1301 in Asian populations is warranted before dapsone therapy (4, 5). Here we report a case of LABD in which the condition was not well controlled under the combination treatment of steroids and sulfasalazine. During the waiting period for HLA genotypes, dupilumab was successfully used to control severe pruritus in the patient. To our knowledge, this is the first report of the application of dupilumab in LABD for adults.

A 52-year-old man was admitted to our clinic with a history of pruritic bullae for 1 month. The lesion initially occurred in his lower extremities, subsequently, it gradually developed to the face and trunk without any mucosal involvement. He was treated with acyclovir for 1 week at the local hospital, yet without amelioration. He had a medical history of hypertension and no family or psychosocial history. Physical examination showed diffuse tense bullae or vesicles containing serous or bloody fluid on the erythematous edematous base. Parts of the blisters arranged as the ‘string of pearls’. Lesions were distributed throughout his face, trunk, limbs, and hands (**Figures 1A, B**). Oral and genital mucous membranes were not involved, superficial lymph nodes were not enlarged, and no positive findings were found in systematic physical examination. A biopsy was performed which revealed a subepidermal blister with neutrophilic infiltrates and lymphocyte and neutrophil infiltration around the superficial vessels in the dermis (**Figure 2**). DIF revealed linear IgA deposits in the BMZ (Figure not shown), confirming the clinical suspicion of LABD. We also performed serum indirect immunofluorescence, which showed Dsg1, Dsg3, BP180 and BP230 were negative. LABD should be distinguished from bullous pemphigoid (BP) and dermatitis herpetiformis (DH). They all belong to autoimmune subepidermal blister disease and appeared as erythema and tense blisters or bullae with severe pruritus. H&E staining usually manifests as subepidermal blisters with infiltration of inflammatory cells in the dermis. BP180 and/or BP230 antibodies are always positive in BP. Diagnosis usually depends on DIF. LABD shows a linear distribution of IgA in the BMZ, BP manifests as linear C3 and/or IgG in BMZ and DH was characterized by granular IgA deposits in the dermal papillary layer.

**Figure 1:**
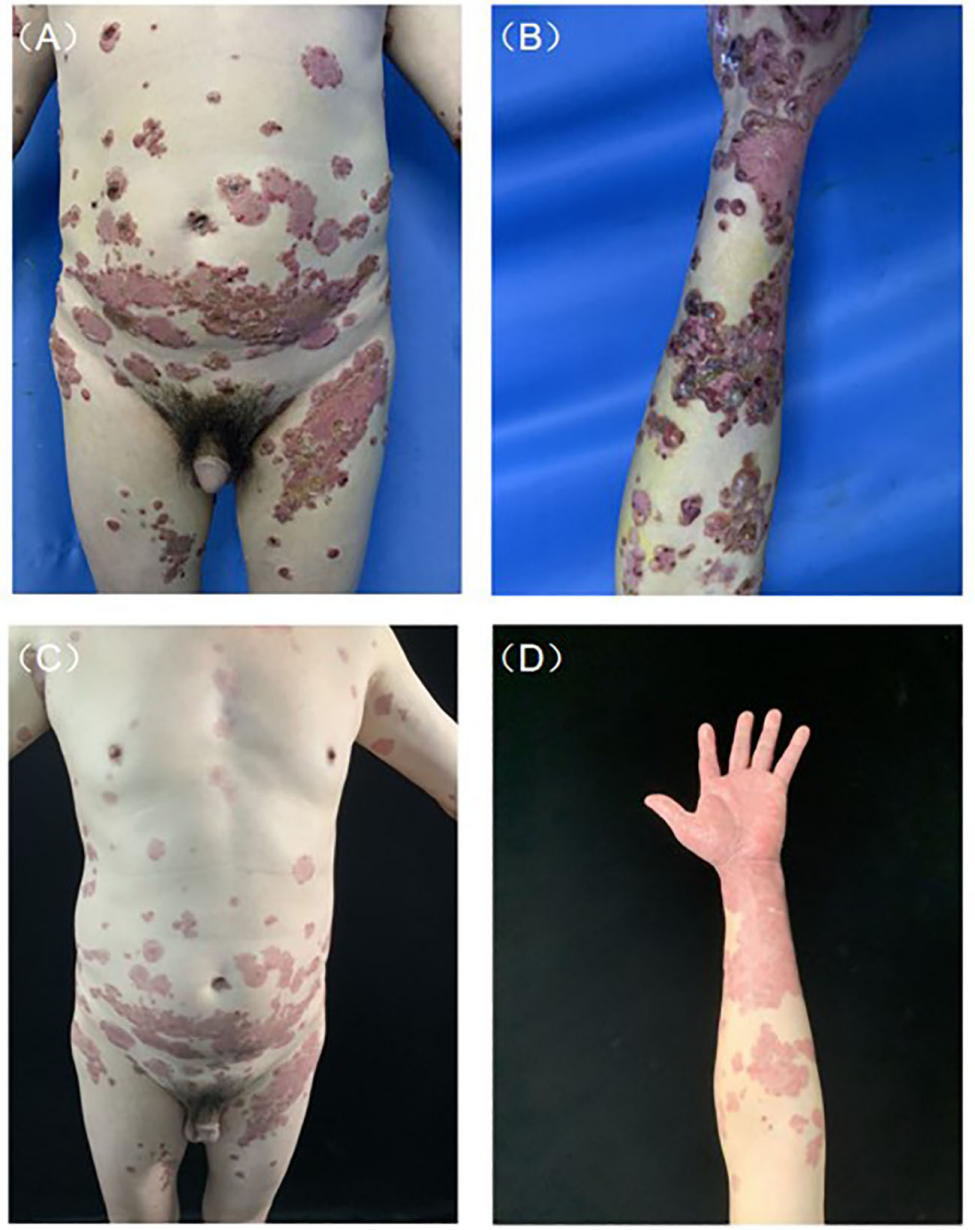
Clinical presentation before and after treatment. **(A)** Typical lesions of tense blisters arranged as the ‘string of pearls’ on the abdomen. **(B)** Diffuse tense bullae or vesicles containing serous or bloody fluid on edematous erythematous base. **(C, D)** After 1 month of treatment, bullae and erosion were removed.

**Figure 2:**
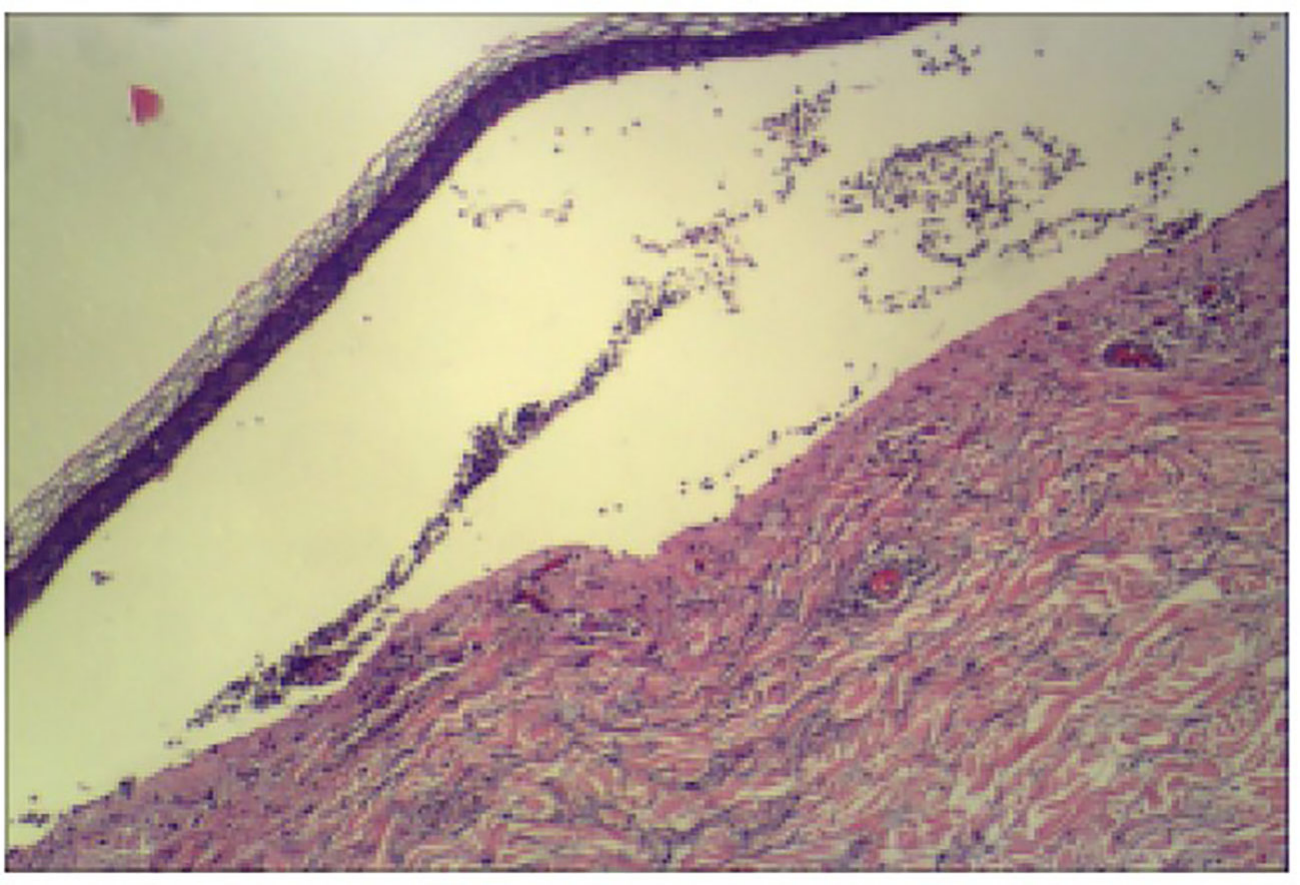
Representative image (40×) of hematoxylin and eosin staining. Biopsy showing a subepidermal blister with neutrophilic infiltrates and lymphocyte and neutrophil infiltration around superficial vessels in the dermis.

Genetic screening for HLA-B*1301 was performed, medium-dose corticosteroid therapy (methylprednisolone 40 mg day^-1^) was started, after three days of treatment, the patient still had more than 10 new bullae and experienced intense itching every day. Sulfasalazine was added at a dose of 1 g day^-1^. After another three days of treatment, severe pruritus had not improved and the patient could not fall asleep all night with peak pruritus numerical rating scale (NRS) reaching 10. Before the results of genetic screening came out, 600 mg dupilumab was administered subcutaneously, the pruritus was significantly relieved the next day, and the patient was finally able to fall asleep peacefully with peak pruritus NRS declined to 3. The results of the genetic tests indicated that HLA-B*1301 was negative and dapsone was applied with a dose of 100 mg day^-1^. The timeline of his treatment course is included in **Table 1**. After 1 month of treatment, rash and severe pruritus were well controlled (**Figures 1C, D**).

**Table 1:** Timeline of the disease status, BPDAI and corresponding therapy in our case.

Time	Disease status	BPDAI	NRS	Therapy
2023-7-30	Edematous erythema, blisters and bullae on the hands, feet and abdomen	97	8	MP 40 mg/d
2023-8-02	more than 10 new bullae everyday, pruritus got worse	115	10	MP 40 mg/d+SASP 1 g/d
2023-8-05	8-10 new blisters everyday, pruritus significantly relieved	128	6	MP 40 mg/d+SASP 1 g/d+Dupi 600 mg
2023-8-15	3-5 new blisters everyday, pruritus reduced	122	4	MP 40 mg/d+DDS 100 mg/d
2023-9-15	bullae and erosion were removed, erythema remained in the original site	63	2	Prednisone 30 mg/d+DDS 100 mg/d

BPDAI, Bullous Pemphigoid Disease Area Index, MP, methylprednisolone, SASP, Sulfasalazine, Dupi, dupilumab, DDS, dapsone.

LABD is a rare autoimmune subepithelial vesicobullous disease caused by IgA autoantibodies, which affects both children and adults. Systemic dapsone is the most common first-line treatment, other alternatives include systemic glucocorticoids, sulfonamides, intravenous immunoglobin, arituximab, etc (6). It is imperative to screen for HLA-B*1301 before initiating dapsone as HLA-B*1301 is associated with DHS in Asian (4).

Dupilumab is a fully human monoclonal antibody blocking the shared receptor component for interleukin-4 and interleukin-13, thus reducing the formation of downstream cytokines (7). Dupilumab is approved for the treatment of atopic dermatitis, asthma and other bullous dermatoses, namely, BP (8, 9). LABD shares many characteristics with BP. Clinically, both are tension blisters on edematous erythema, usually accompanied by severe itching. Pathologically, they are all epidermal blisters with inflammatory cell infiltration. Most patients with LABD have IgA antibodies targeting the 97 kDa and 120 kDa antigens in the BMZ, both of these antigens are extracellular fragments of bullous pemphigoid antigen 2 (BP180) (1, 10). In rare cases, the NC16A domain of BP180 is also founded in LABD (11). It has been postulated that LABD represents an IgA response to BP immune antigens. So we attempted to use dupilumab to treat LABD and achieved a good response. To the best of our knowledge, only 2 cases of LABD in children have been successfully treated with dupilumab to date (12, 13).

In this case, the patient did not respond well to corticosteroids combined with sulfasalazine and dupilumab was applied to relieve severe pruritus before the results of the genetic test were released and received a good response. This study suggests that dupilumab may be an alternative option for LABD patients with contraindications of dapsone or resistance to conventional treatment. However, the actual mechanism of dupilumab in LABD is not fully understood. Immune profiles in the lesion and the peripheral blood of LABD should be elucidated and clinical studies will be conducted to confirm its effectiveness in the future.

## Data availability statement

The original contributions presented in the study are included in the article/supplementary material. Further inquiries can be directed to the corresponding author.

## Ethics statement

Written informed consent was obtained from the individual(s) for the publication of any potentially identifiable images or data included in this article.

## Author contributions

LW: Writing – original draft, Resources. JP: Writing – review & editing. JC: Writing – review & editing, Validation, Funding acquisition, Conceptualization.
